# Kikuchi Disease in Children: A Report of Two Cases

**DOI:** 10.7759/cureus.35160

**Published:** 2023-02-18

**Authors:** Motaz Azzam, Hadi Helali, Elsade Sharif, Rehab Musa, Moataz Hamdi, Hassan Hotait, Mohammed Aldirawi, Sarmad Alhamdani, Lemis Yavuz

**Affiliations:** 1 Pediatrics, Al Jalila Children's Speciality Hospital, Dubai, ARE; 2 Pediatric Rheumatology, Al Jalila Children's Speciality Hospital, Dubai, ARE; 3 Hospital Medicine, Al Jalila Children's Speciality Hospital, Dubai, ARE; 4 Pathology, Dubai Health Authority, Dubai, ARE

**Keywords:** necro-inflammation, prolonged fever, neck lymph adenitis, necrotizing lymphadenitis, kikuche disease

## Abstract

Kikuchi disease (KD) is a benign self-limiting rare disease with unknown etiology. Prolonged fever with tender neck lymphadenitis is the most common presentation. Blood tests are not specific, and the final diagnosis is by biopsy. We describe two patients, ages seven and twelve years, who presented with fever and neck lymphadenitis. Both cases received antibiotics for more than two weeks without improvement. Blood work showed high inflammatory markers. The manifestation of the second case overlapped with Hashimoto's disease. The later diagnosis was confirmed by lymph node (LN) biopsy.

## Introduction

Kikuchi disease (KD) is a challenging disease. It causes necrotizing lymphadenitis and mimics other diseases such as tuberculosis, lymphoma, or systemic lupus erythematosus (SLE) [1,2]. It was considered an adult disease, hence patients from the pediatric age group were exposed to extra workup or unnecessary treatment [3].

Definite diagnosis is by excisional lymph node (LN) biopsy with a specific histology finding. The treatment is supportive, and recovery is expected in the majority of cases. Follow-up for recurrence is important. Medical records reported a few patients who developed autoimmune diseases later in life, such as SLE or Hashimoto's disease [1].

We describe two patients, ages seven and 12 years, who presented with lymphadenitis. Both received antibiotics for a long time without improvement. Their initial investigation showed high inflammatory markers. The manifestation of the second case overlapped with Hashimoto's disease. The final diagnosis was confirmed by LN biopsy.

## Case presentation

Case 1

A previously healthy six-year-old girl presented at Al Jalila Children's Speciality Hospital in August with a one-month history of on-and-off fever of unknown origin and swollen and tender cervical lymphadenopathy. She was in her usual state of health until the end of July 2022, when she started to have the symptoms mentioned above, for which she was taken to another facility. Her investigations initially showed high inflammatory markers and a negative strep test. She received amoxicillin-clavulanic acid orally (45mg/kg/day of amoxicillin) for eight days without improvement.

Further tests were done, which showed persistently elevated C-reactive protein (CRP). Neck and abdomen ultrasound (US) studies showed inflamed cervical lymph adenitis and hepatosplenomegaly. A neck computed tomography (CT) scan was reassuring. Then she received IV ceftriaxone (75 mg/kg/day) for three days, then oral cefixime (8mg/kg/day) for seven days. She lost 1 kg during that period, and her symptoms were associated with fatigue and decreased appetite.

As a result of persistent symptoms, the child was admitted to our hospital in September. The admission examination showed a supple neck, with right anterior cervical LN 3 x 3 cm, mobile and non-tender, left anterior cervical LN 2 x 2 cm, multiple right and left axillary LN less than 1cm, bilateral small inguinal LN, all mobile and non-tender. Otherwise, her physical examination was normal, apart from an erythematous skin rash. The initial blood workup was inconclusive (Table 1).

**Table 1:** Initial Blood Investigations. WBC: white blood cell, Hgb: hemoglobin, MCV: mean corpuscular volume, MCH: mean corpuscular hemoglobin, PCR: polymerase chain reaction, CMV: cytomegalovirus, EBV: Epstein-Barr virus, HHV-6: human herpesvirus-6.

Investigation	Results	Reference range
Complete Blood Count		
WBC	6.46 x 10^3^/mcl	4.00–11.00x10^3^
Hgb	10.3 gm/dL	12.0–15.0
MCV	77.3 fL	80.00–100.00
MCH	25.10 pg	27.00–32.00
Platelet	575,000/mcl	150.00–450.00
Neutrophil %	19.7%	40.00–80.00
Lymphocyte %	58.4%	18.00–42.00
Neutrophil Absolute	1.28 x10^3^/mcl	2.00–7.00
Lymphocyte Absolute	3.77 x10^3^/mcl	1.00–4.00
Inflammatory Markers		
C-Reactive Protein	94 mg/L	0.0–5.0
Erythrocyte Sedimentation Rate	107 mm/hr	0–10
*Lactate dehydrogenase*	544 U/L	0–300
Ferritin	175ng/ml	13.8–78.8
Blood Culture	Negative	-
Respiratory panel PCR	Negative	-
COVID-19 PCR swab	Negative	-
CMV, EBV, HHV6	Negative	
Blood film	Mild normocytic normochromic anemia/relative neutropenia and reactive picture.	
T-spot	Negative	
Brucella IgG, IgM	Negative	
Direct Coombs	IgG Positive, C3 Negative	
ANA	Nucleosomes positive, otherwise negative	

**Figure 1:**
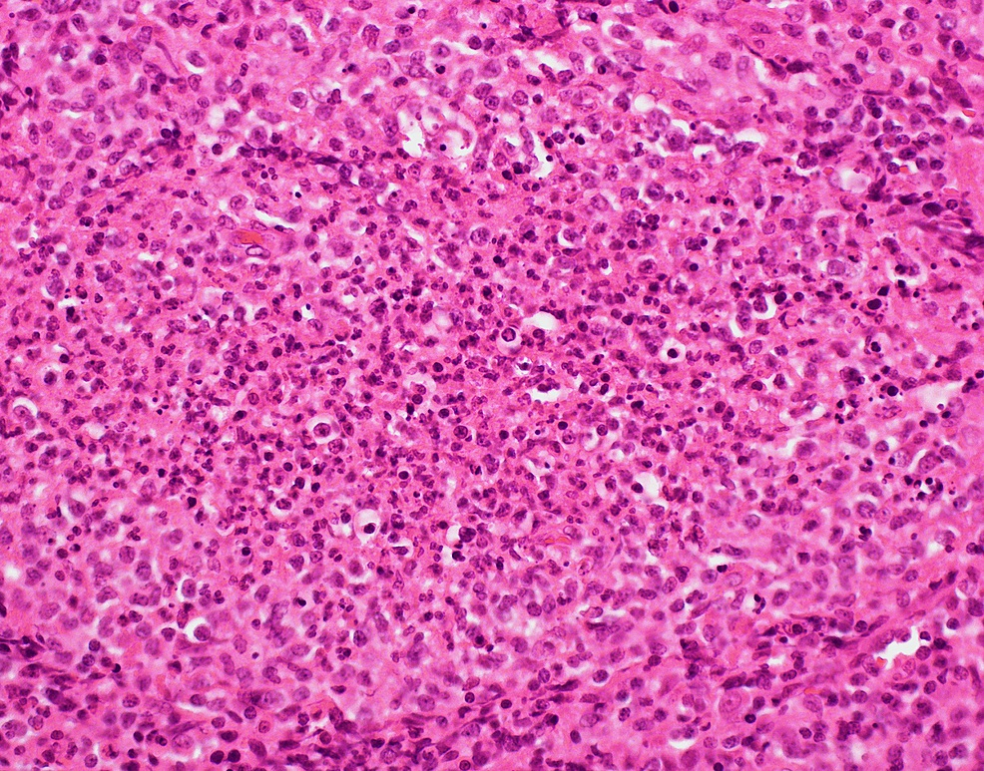
Neck lymph node biopsy: ematoxylin and eosin-stained sections prepared from formalin-fixed paraffin-embedded tissue from excised representative lymph nodes. CD3 (T-cell immunomarker) and CD68 (histiocytic immunomarker) immunohistochemistry were performed and showed T-lymphocytic and histiocytic infiltrate in the necrotic areas.

A neck US revealed multiple enlarged LN in the cervical and submandibular region. One of them measured 1.8 x 1.5 cm. On US Doppler interrogation, internal vascularity was seen. There was no evidence of suppuration or adjacent surrounding inflammatory changes. Given this clinical picture, the differential diagnosis included infectious lymphadenitis, autoimmune causes such as SLE, and malignancy, particularly lymphoma. Hence, the decision was made to do an LN biopsy (Figure 1).

The excised LN showed massive areas of eosinophilic necrotic tissue, replacing the nodal architecture. They consist of necrotic debris material intermixed with karyorrhexis, lymphocytes, and histiocytes with crescentic nuclei (features suggestive of a well-established necrotizing stage). Neutrophils, eosinophils, plasma cells, giant cells, epithelioid granulomas, and viral cytopathic changes were not identified. The clinical and histological findings are consistent with KD.

Given the nature of the disease and the child's stable condition, she was discharged home with Naproxen 5mg/kg/dose twice daily for ten days. On follow-up in the clinic two weeks later, the LNs had decreased in size to less than 0.5 cm, inflammatory markers normalized, and the child was asymptomatic.

Case 2

A previously healthy 12-year-old girl presented with a history of neck swellings for one month and a high-grade fever for two weeks. She was in her usual state of health until the middle of March 2022, when she started complaining of neck swellings and pain. The swellings were bilateral and painful, especially on manipulation. Two weeks later, the girl began to develop a high-grade fever, reaching 39.5 Celsius, associated with generalized body aches and erythematous skin rash. The patient received 10 days of oral amoxicillin-clavulanate acid, 45mg/kg/day. The neck swellings did not change in size during this period. So, she was admitted to our hospital.

The physical examination was positive for tender neck lymphadenitis and erythematous skin rash. She received a course of intravenous clindamycin for 10 days (40 mg/kg/day). Blood tests showed leukopenia with deranged thyroid tests, and the results of thyroid antibodies were high (antithyroglobulin 59.25 IU/mL and the thyroid peroxidase (TPO) antibodies 1,949.79 IU/mL) (Table 2).

**Table 2:** Initial Blood Investigations. WBC: white blood cell, Hgb: hemoglobin, MCV: mean corpuscular volume, MCH: mean corpuscular hemoglobin, TSH: thyroid stimulating hormone.

Investigation	Results	Reference range
Complete Blood Count		
WBC	2.57	4.00-11.00
Hgb	10.1 gm/dL	12.0–15.0
MCV	69.60 fL	80.00–100.00
MCH	21.50 pg	27.00–32.00
Platelet	178,000	150.00–450.00
Neutrophil %	38.90%	40.00–80.00
Lymphocyte %	52.50%	18.00–42.00
Neutrophil Absolute	1.00 x10^(3)^	2.00–7.00
Lymphocyte Absolute	1.35 x10^(3)^	1.00–4.00
Inflammatory Markers		
C–Reactive Protein	2.4 mg/L	0.0–5.0
Erythrocyte Sedimentation Rate	13 mm/hr	0–10
*Lactate dehydrogenase*	581	0–300
Thyroid function test		
T4	12.4 pmol/L	12.6–21.0
TSH	4.41 mIU/L	0.51–4.30

**Figure 2:**
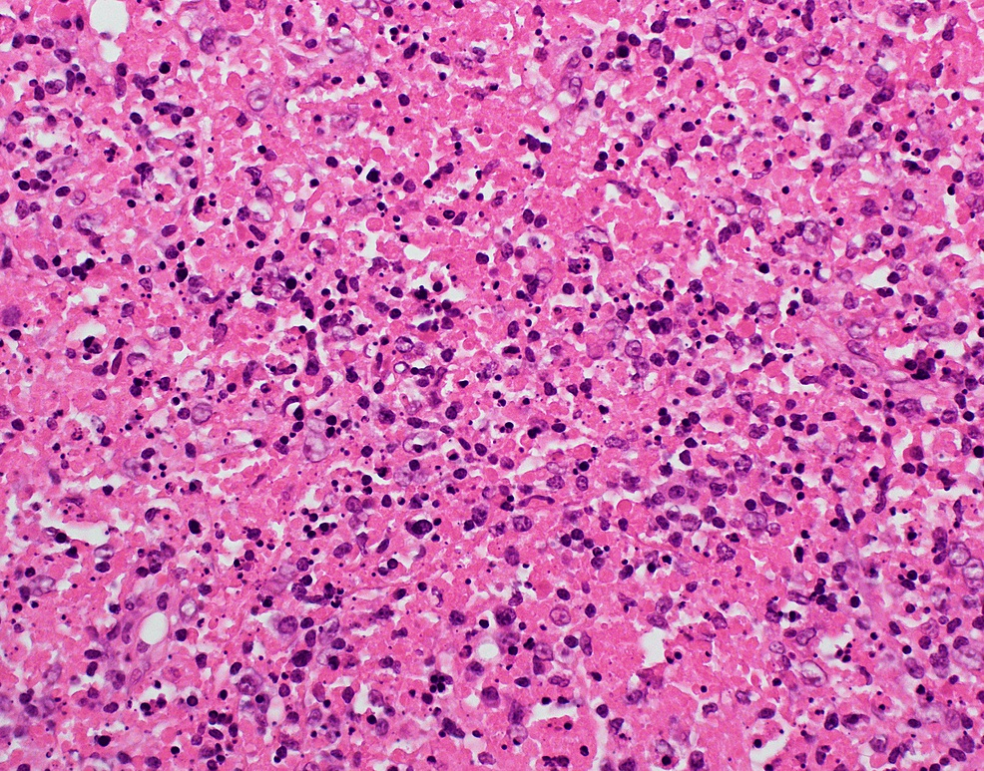
Lymph node biopsy showed areas of eosinophilic necrotic debris with nuclear dust (karyorrhexis) intermixed with T-lymphocytes and crescentic histiocytes without neutrophils, eosinophils, plasma cells, and epithelioid granuloma.

She was diagnosed with Hashimoto's thyroiditis and was started orally on L-Thyroxin 25 mcg once daily. Meanwhile, she continuously had high-grade fevers every day of admission, reaching 39.5 degrees. Her LN swellings did not reduce in size, and they were still painful. Therefore, additional workup was done. Screening for SLE came back negative, apart from a weekly positive antinuclear antibody test (ANA) titer. A neck US showed the thyroid gland with multiple hypoechoic spots suggestive of thyroiditis (Hashimoto). Bilaterally, the neck showed multiple enlarged LN. An excisional LN biopsy and bone marrow (BM) also were done. The BM was normal, and the excisional LN biopsy showed focal areas of eosinophilic necrotic debris of karyorrhexis, lymphocytes, and histiocytes. The background showed reactive lymphoid follicles and mottled interfollicular areas (features suggestive of the early proliferative stage). In addition, the immunohistochemistry marker profile was in keeping with Kikuchi's Histiocytic Necrotizing Lymphadenitis (CD68: Diffusely positive in the paracortical and medullary area, CD3: Numerous T-cells are positive, CD20: Lymphoid follicles only positive, CD30 and CD15: Negative). So, the child was diagnosed with Kikuchi's Histiocytic Necrotizing Lymphadenitis (Figure 2).

On day 11 of admission, the patient started to become afebrile, and her activity level returned to normal. In addition, her LNs started to decrease in size. She was discharged home on regular oral Ibuprofen 10 mg/kg/dose twice daily for seven days and on L-Thyroxin 25 mcg orally. On examination after two months, she was asymptomatic and doing well.

## Discussion

KD, also known as necrotizing lymphadenitis, was first described in Japan in 1972. The etiology is unknown. However, theories suggest an autoimmune response to a trigger-like infection as the probable cause of this disease [4]. Few reports in the literature document a link between autoimmune diseases such as Hashimoto's disease or SLE and KD [5]. Follow-ups done on 23 Asian patients reported KD as the first manifestation of SLE in one patient [6]. Moreover, the second case in this report was diagnosed with Hashimoto on presentation.

KD can affect all age groups; however, it has been underdiagnosed in children. Initially, it was thought to be a disease in young adults, but then the literature started to recognize it in children [3]. The mean age group is 21.5 ± 11.8 years, with a predominance of males in patients aged ≤ 9 years.

This is a challenging disease because the signs and symptoms overlap with other illnesses. The most common presentations in KD are prolonged fever, tender neck lymphadenopathy, and skin rash [7]. It is sometimes associated with leukopenia and increased inflammatory markers. These clinical and laboratory findings can result in a misleading diagnosis of autoimmune disease, infection, or lymphomas. Hence, being aware of KD as a possible diagnosis is important so that patients are not exposed to extra workups or treatment [8].

Diagnosis can be done by LN biopsy. Blood results and image studies are inconclusive. Fine needle aspiration has a high rate of false negative results. It was accurate in 56.25% of cases in a retrospective study done on 44 patients with KD [9]. An excisional LN biopsy with specific histological findings is the preferable test to reach the diagnosis and define the stage. Immunohistochemical studies help in differentiating KD from other illnesses such as lymphoma or SLE.

 The outcome of KD is excellent, and spontaneous recovery is expected with supportive treatment only. Given the active inflammatory process during the active phase, improvement is expected by using nonsteroidal anti-inflammatory drugs (NSAID). Our two cases recovered fully on Naproxen and Ibuprofen. Still, close observation during the illness is required, as rare complications such as neurological lesions have been documented [10]. Additionally, follow-up is essential as recurrent symptoms have been described. A few cases developed SLE or Hashimoto's disease later in life [5,11].

## Conclusions

KD is necrotizing lymphadenitis. Although it is a benign and self-limiting disease, complications and recurrent symptoms have been reported. The clinical presentations mimic other severe illnesses, such as SLE or lymphoma, that lead to extra workup and treatment or sometimes misleading diagnoses. More studies and follow-ups are needed on this disease to identify the etiology and to predict the risk factor of complications and recurrence. 
